# Integrating Apparent Diffusion Coefficient and Prostate-Specific Antigen as Prognostic Factors of Treatment Response to Androgen Deprivation Therapy and Radiotherapy in Prostate Cancer

**DOI:** 10.3390/biomedicines13081979

**Published:** 2025-08-15

**Authors:** Victor Duque-Santana, Julio Fernandez, Fernando López-Campos, Ana Diaz-Gavela, Manuel Recio, Luis L. Guerrero, Marina Peña, Sofia Sanchez, Israel J. Thuissard, Cristina Andreu-Vázquez, David Sanz-Rosa, Giulia Marvaso, Alfonso Gómez-Iturriaga, Thomas Zilli, Elia Del Cerro, Felipe Couñago

**Affiliations:** 1 Department of Radiation Oncology, Hospital Universitario Quirónsalud Madrid y Hospital La Luz, 28223 Madrid, Spain; anadiazgavela@gmail.com (A.D.-G.); luisleoguerrero01@gmail.com (L.L.G.); marina.pena@quironsalud.es (M.P.); sofia.sanchez@quironsalud.es (S.S.); elia.delcerro@quironsalud.es (E.D.C.); 2 Department of Medicine, Faculty of Medicine, Health and Sports, European University of Madrid, 28108 Madrid, Spain; manuel.recio@quironsalud.es (M.R.); israeljohn.thuissard@universidadeuropea.es (I.J.T.); david.sanz@universidadeuropea.es (D.S.-R.); felipe.counago@genesiscare.es (F.C.); 3 Department of Radiology, Hospital Universitario Quirónsalud Madrid, 28223 Madrid, Spain; julio.fernandezma@quironsalud.es; 4 Department of Radiation Oncology, Hospital Universitario Ramon y Cajal, 28034 Madrid, Spain; fernando_lopez_campos@hotmail.com; 5 Department of Veterinary Medicine, Faculty of Biomedical and Health Sciences, European University of Madrid, 28108 Madrid, Spain; cristina.andreu@universidadeuropea.es; 6 Division of Radiation Oncology, IEO European Institute of Oncology, IRCCS, 20141 Milan, Italy; giulia.marvaso@ieo.it; 7 Department of Oncology and Hemato-Oncology, University of Milan, 20122 Milan, Italy; 8 Department of Radiation Oncology, Hospital Universitario de Cruces, 48903 Barakaldo, Spain; agomeziturriaga@gmail.com; 9 Department of Radiation Oncology, Oncology Institute of Southern Switzerland, EOC, 6500 Bellinzona, Switzerland; thomas.zilli@eoc.ch; 10 Faculty of Biomedical Sciences, Università della Svizzera italiana, 6900 Lugano, Switzerland; 11 Faculty of Medicine, University of Geneva, 1206 Geneva, Switzerland; 12 Department of Radiation Oncology, GenesisCare, Hospital San Francisco de Asís y La Milagrosa, 28002 Madrid, Spain

**Keywords:** radiotherapy, apparent diffusion coefficient, PSA, androgen deprivation therapy, prostate cancer

## Abstract

**Purpose:** This study evaluates the combined prognostic value of the apparent diffusion coefficient (ADC) from multiparametric MRI (mpMRI) and prostate-specific antigen (PSA) levels at 6 months post-radiotherapy (RT) in assessing treatment response in prostate cancer patients treated with RT and androgen deprivation therapy (ADT). **Materials and Methods:** All prostate cancer patients classified as unfavorable intermediate-risk, high-risk, or very high-risk, according to NCCN criteria, who received ADT and RT between 2008 and 2019 and underwent mpMRI and PSA testing 6 months after RT were included. Patients were stratified into three profiles based on threshold PSA (≤ vs. >0.1 ng/mL) levels and ADC (≤ vs. >1.24 × 10^−3^ mm^2^/s) values: Profile A: low PSA and high ADC; Profile B: either high PSA/high ADC or low ADC/low PSA; Profile C: high PSA and low ADC. Ten-year progression-free survival (PFS) and metastasis-free survival (MFS) were analyzed using Kaplan–Meier curves and multivariate Cox regression. ** Results:** Ninety-eight consecutive patients were retrospectively analyzed, of which 73 (74.5%) were high-risk. After a mean follow-up of 95.3 months, 19 (19.39%) patients progressed. Ten-year PFS, MFS, and overall survival were 75.6%, 87%, and 89.5% respectively. Progression events were 9.1% (Profile A), 29.4% (Profile B), and 44.4% (Profile C). Eight-year PFS was 89.2% (profile A), 70.9% (profile B) HR: 3.021 (CI 95%: 1.031–8.849; *p* = 0.044) and 44.4% (profile C) HR: 6.145 (CI 95%: 1.645–22.955; *p* = 0.007). Multivariate analysis confirmed a higher risk of progression in patients from profile B with HR: 3.958 (CI 95%: 1.18–13.191; *p* = 0.025) and profile C with HR: 41.945 (CI 95%: 5.000–351.761; *p* < 0.001) compared to patients from profile A. Metastasis events were 5.5% (Profile A), 8.8% (Profile B), and 33.3% (Profile C). Eight-year MFS was 100% (profile A), 89.6% (profile B) HR: 1.373 (CI 95%: 0.277–6.811; *p* = 0.689), and 74.1% (profile C) HR: 5.566 (CI 95%: 1.119–27.692; *p* = 0.047). **Conclusions:** The integration of PSA response and ADC measures at 6 months post-RT provides an effective combined prognostic factor to identify patients at higher risk of relapse, supporting closer monitoring and potential treatment intensification.

## 1. Introduction

Prostate cancer is the second most commonly diagnosed malignancy worldwide [1]. Treatment options for localized intermediate- and high-risk disease include radiotherapy (RT) with or without androgen deprivation therapy (ADT), or radical prostatectomy, all of which yield comparable oncological outcomes [2].

In recent years, significant progress has been made not only in refining treatment strategies, but also in advancing the shift toward precision medicine. This approach aims to identify patients with an unfavorable prognosis and a higher risk of recurrence who may benefit from intensified treatment and closer monitoring, while sparing patients with more favorable characteristics from unnecessary interventions.

In alignment with this goal, considerable efforts have been dedicated to identifying biomarkers that can predict treatment response. Notably, recent studies have focused on biochemical markers such as prostate-specific antigen (PSA) levels measured 6 months after the completion of radiotherapy. For example, a study by Kwak et al. [3], published in 2024, analyzed PSA levels at 6 months post-RT across 16 clinical trials. Their findings showed that patients with PSA levels > 0.1 ng/mL had significantly worse treatment outcomes compared to those with lower PSA levels.

Simultaneously, the use of multiparametric magnetic resonance imaging (mpMRI) has become widespread in the diagnosis, staging, and follow-up of prostate cancer [4].

Among the parameters derived from mpMRI, the apparent diffusion coefficient (ADC), obtained from diffusion-weighted imaging (DWI) sequences, provides quantitative information on tissue cellularity and has been associated with tumor detection, characterization, and staging in prostate cancer [5,6,7].

The potential role of post-RT ADC values as biomarkers of treatment response was initially reported by Liu et al. in 2014 [8]. More recently, in our previously published study (2025) [9], we validated these findings, demonstrating that ADC values assessed 6 months after RT are associated with outcomes in prostate cancer patients. An ADC cutoff of 1.24 × 10^−3^ mm^2^/s was identified for stratifying risk of disease progression.

Although biochemical (PSA) and radiological (ADC) markers have independently been associated with prognosis following RT and ADT, the combined prognostic value of these two parameters has not yet been explored.

The integration of biochemical and radiological markers, such as PSA and ADC, could represent a promising approach in the refinement of prognostic models in prostate cancer. PSA reflects systemic tumor activity, while ADC provides quantitative imaging data related to local tissue cellularity. Their combined assessment may enhance the identification of patients with adverse biological behavior, supporting risk-adapted management strategies following RT and ADT.

The objective of the present study is to investigate whether integrating biochemical response (PSA) and radiological (ADC) markers, both assessed 6 months post-RT, could identify prostate cancer patients at increased risk of relapse following combined ADT and RT.

## 2. Materials and Methods

### 2.1. Patient Selection

The protocol for this study was approved by the ethics committee at Quironsalud University Hospital on 14 November 2023 (EO277-23_HUQM). This was a retrospective study of patients diagnosed with unfavorable intermediate-risk (IR), high-risk, or very high-risk prostate cancer (HRP) treated with RT and ADT with curative intent between 2008 and 2019, with availability of 6-month post-RT mpMRI scans and sufficient data available for calculating tumor ADC values and PSA levels obtained 6 months after the end of RT.

Exclusion criteria included initial treatment with radical prostatectomy; patients classified as having low-risk or favorable intermediate-risk prostate adenocarcinoma (due to the distinct biological characteristics of these patients, who generally exhibit a more favorable prognosis, and the absence of ADT in this subgroup, in order to achieve an homogeneous study population); not receiving combined RT and ADT; not undergoing mpMRI after completion of RT; inability to calculate tumor ADC values; and patients without PSA determination at 6 months after RT.

All patients had a confirmed diagnosis of prostate adenocarcinoma based on transrectal ultrasound-guided biopsy. Gleason scores and PSA levels were available for all cases. Clinical staging included digital rectal examination and transrectal ultrasound performed by experienced urologists at our institution. All patients underwent a baseline mpMRI. Based on these findings, patients were stratified into NCCN risk categories.

Patients diagnosed with intermediate- or high-risk prostate cancer underwent additional staging with computed tomography of the chest, abdomen, and pelvis (CT-CAP) and bone scintigraphy.

Patients were stratified into three profiles based on established thresholds derived from previous study results: PSA (0.1 ng/mL) levels from Kwak et al. [3] and ADC (1.24 × 10^−3^ mm^2^/s) values from Duque-Santana V et al. [9].

- **Profile A**: low post-RT PSA (≤0.1 ng/mL) and high post-RT ADC (>1.24 × 10^−3^ mm^2^/s)
- **Profile B**: either high post-RT PSA (>0.1 ng/mL) with high ADC (>1.24 × 10^−3^ mm^2^/s), or low post-RT ADC (≤1.24 × 10^−3^ mm^2^/s) with low PSA levels (≤0.1 ng/mL)
- **Profile C**: high post-RT PSA (>0.1 ng/mL) and low post-RT ADC (≤1.24 × 10^−3^ mm^2^/s)

### 2.2. Treatment

All patients received intensity-modulated radiotherapy (IMRT) and daily image-guided radiation therapy, with no fiducial placement, with total doses and fractionation schedules adapted to their risk group. Between 2009 and 2013, IR and HRP patients received 80 Gy delivered in 2 Gy fractions. After a protocol change in 2013, patients received 70.2 Gy in 2.7 Gy fractions (equivalent to 80.03 Gy EQD2).

The clinical target volume (CTV) for patients with intermediate- and high-risk disease confined to the prostate (stage T1–T2) included the prostate; additionally, for intermediate risk, the CTV included the base of the seminal vesicles, and for high-risk, the CTV included the entire seminal vesicle area. The corresponding volume for patients with locally advanced disease (T3–T4) extended beyond the prostate to cover extracapsular extension [10]. None of the patients received whole-pelvis radiotherapy.

ADT duration varied according to risk classification: HRP patients received long-term ADT (24 months), while IR patients received short-term ADT (6 months). All patients initiated neoadjuvant ADT 2 months before starting RT, according to institutional protocols.

### 2.3. Follow-Up Procedure

Patients were monitored every 3 months during the first 2 years, every 6 months during the following 3 years, and annually thereafter. Follow-up evaluations included clinical history, physical examination, and laboratory testing (including PSA levels).

Post-RT mpMRI was performed at 6 months to assess treatment response and to obtain ADC measurements (technical details on mpMRI have been previously published [9]). A PSA test was also obtained at 6 months post-RT.

Biochemical recurrence was defined according to the Phoenix criteria (PSA nadir + 2 ng/mL). In the event of biochemical recurrence, patients underwent prostate mpMRI and CT of the chest, abdomen, and pelvis, along with bone scintigraphy or choline/PSMA PET to detect local, locoregional, or distant relapse.

- If mpMRI and conventional imaging suggested exclusive local recurrence, confirmation by prostate biopsy was required.
- If locoregional or distant relapse was suspected based on conventional imaging, choline/PSMA PET was used for confirmation.

### 2.4. Image Analysis

Two radiologists with more than 25 and 15 years’ experience with MRI in the diagnosis of genitourinary disorders determined in consensus the location of each tumor using the Prostate Imaging Reporting and Data System. MRI findings for prostate cancer included a focal lesion with high signal intensity on T2-weighted images, which showed low signal intensity on the ADC map and high signal intensity on DW images with a *b*-value of 1000 mm^2^/s, with or without early contrast enhancement and rapid washout on dynamic contrast-enhanced imaging. ADC maps were generated pixel by pixel using the integrated software tool Functool version 2.1. Regions of interest containing as much of the tumor as possible were drawn onto the ADC maps. For tumors visible in multiple slices, ADC values were calculated for each slice, and for patients with multiple lesions, all the lesions were measured. In both cases, the lowest values were taken. For the post-RT ADC calculations, both radiologists in the consensus drew a region of interest at the site of each original tumor. The calculations were made using the same methods described above.

### 2.5. Statistical Analysis

Statistical analyses were performed in IBM SPSS Statistics version 21.0 (IBM Corp., Armonk, New York, NY, USA).

Biochemical recurrence-free survival (bRFS), local recurrence-free survival (LRFS), MFS, PFS, OS, and prostate cancer-specific survival (PCSS) were calculated from the date of diagnosis (prostate biopsy) to the occurrence of the corresponding event.

Eight-year PFS and MFS among these profiles were analyzed using Kaplan–Meier curves. Potential relationships were studied using the t-test or Mann–Whitney U test for quantitative variables and the chi-square or Fisher exact test for qualitative variables.

Multivariate analyses were performed using Cox proportional-hazards regression models incorporating predictive factors related to PFS: age, T stage, risk group, Gleason score, and initial PSA and ADC levels.

## 3. Results

Between 2008 and 2019, 124 patients diagnosed with prostate cancer at our hospital received RT and ADT and had a post-RT mpMRI. Of these, 110 patients had an mpMRI at 6 months after treatment, and 98 had enough data to calculate tumor ADC values and measure PSA levels at that time.

A total of 98 patients met all inclusion criteria and none of the exclusion criteria, so they were included in the final analysis. Of these, 73 (74.5%) were classified as high-risk or very high-risk, and 25 (25.5%) as unfavorable intermediate-risk. The median initial PSA level was 10.15 ng/mL [IQR: 6.93–21], and the mean initial ADC was 0.81 ± 0.18 × 10^−3^ mm^2^/s.

ADT with injectable luteinizing hormone-releasing hormone (LHRH) agonists was administered for a duration of 6 months in 26 patients (26.5%) and 24 months in 69 patients (70.4%). It was discontinued due to poor tolerance in three patients (3.1%) (after 9, 12, and 18 months). All patients received neoadjuvant ADT, started 2 months before radiotherapy.

All patients received IMRT to the target volumes described in the Materials section. The prescribed doses were as follows: 47 patients (47.9%) received 80 Gy in 2 Gy fractions, 48 patients (48.9%) received 70.2 Gy in 2.7 Gy fractions, 2 patients (2.0%) received 76 Gy in 2 Gy fractions, and 1 patient (1.0%) received 78 Gy in 2 Gy fractions. None of the patients received whole-pelvis radiotherapy.

After stratification according to post-RT PSA and ADC thresholds (0.1 ng/mL [3] and 1.24 × 10^−3^ mm^2^/s [9], respectively), 55 patients were assigned to Profile A, 34 to Profile B, and 9 to Profile C. Baseline characteristics were balanced across groups, with no significant differences observed (Table 1).

The PSA values at 6 months post-RT were as follows: Profile A: 0.04 [0.02–0.04], Profile B: 0.07 [0.04–0.12], and Profile C: 0.29 [0.16–0.46]; *p* < 0.001. The ADC results at 6 months post-RT were as follows: Profile A: 1.35 [1.30–1.44], Profile B: 1.20 [1.10–1.36], and Profile C: 1.15 [1.00–1.21]; *p* < 0.001.

After a mean follow-up of 95.36 months (SD: 30.54), 19 (19.39%) patients progressed. For the whole population, 10-year PFS, MFS, and OS were 75.6%, 87%, and 89.5%, respectively.

Progression events according to profile were 9.09% (5/55) in patients with Profile A, 29.41% (10/34) in patients with Profile B, and 44.44% (4/9) in patients with Profile C.

**Figure 1:**
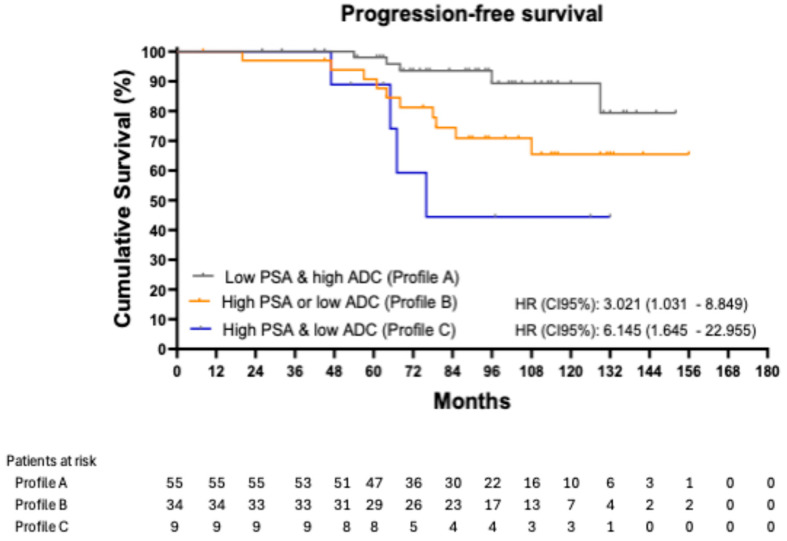
Progression-free survival stratified into three profiles.

Figure 1 shows the Kaplan–Meier curves for PFS, stratified by profile. The 8-year PFS was 89.22% in patients with Profile A, 70.89% in those with Profile B, and 44.44% in those with Profile C. Using patients in Profile A as the reference group, those in Profile B had an HR of 3.021 (95% CI: 1.031–8.849; *p* = 0.044), while those in Profile C had an HR of 6.145 (95% CI: 1.645–22.955; *p* = 0.007).

Metastasis events according to profile were 5.45% (3/55) in patients with Profile A, 8.82% (3/34) in patients with Profile B, and 33.33% (3/9) in patients with Profile C.

**Figure 2:**
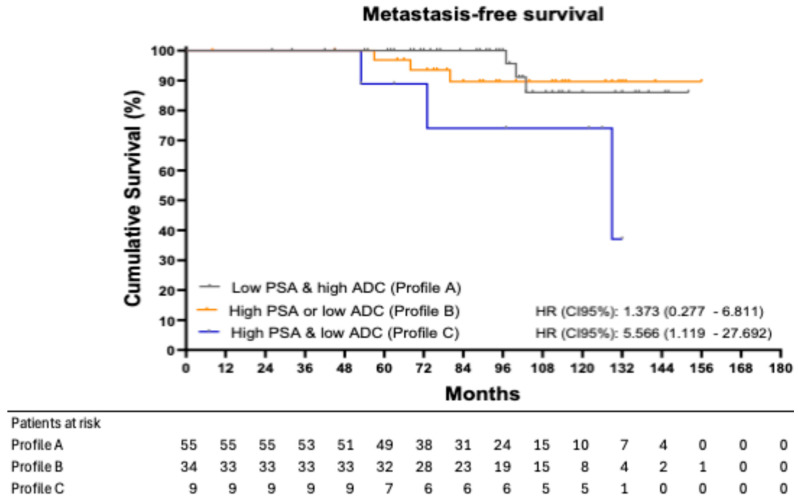
Metastasis-free survival stratified into three different profiles.

Figure 2 shows the Kaplan–Meier curves for MFS, stratified by profile. The 8-year MFS was 100.00% in patients with Profile A, 89.64% in those with Profile B, and 74.07% in those with Profile C. Using Profile A as the reference group, patients in Profile B had an HR of 1.373 (95% CI: 0.277–6.811; *p* = 0.689), while patients in Profile C had an HR of 5.566 (95% CI: 1.119–27.692; *p* = 0.047).

**Figure 3:**
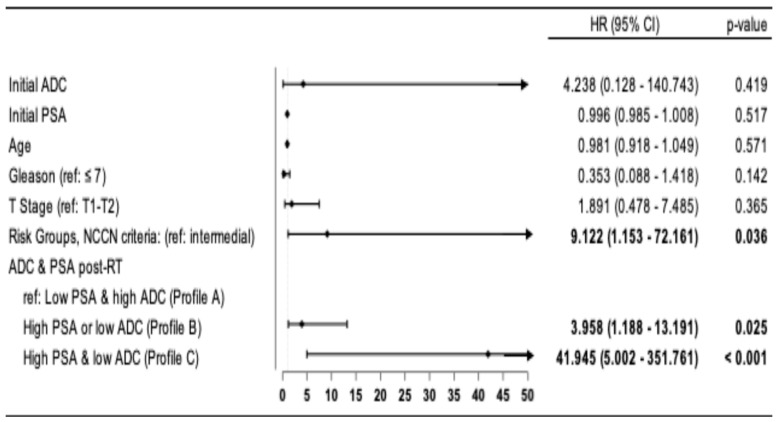
Multivariable analyses of the predictive and prognostic factors for progression-free survival.

In the multivariate analyses, Cox proportional hazards regression models confirm that, after adjustment for covariates (age, T-stage, risk group, Gleason score, and initial PSA and ADC levels), there is a higher risk of progression in patients with Profile B, HR: 3.958 (95% CI: 1.188–13.191; *p* = 0.025) and Profile C, HR: 41.945 (95% CI: 5.000–351.761; *p* < 0.001) compared to patients with Profile A. A higher risk was also observed in HRP patients compared to IR patients, HR: 9.122 (95% CI: 1.153–72.161; *p* = 0.036) (Figure 3).

## 4. Discussion

This study is the first to integrate both biochemical and radiological markers to prognosticate treatment response in prostate cancer patients treated with RT and ADT. Specifically, we demonstrated that combining the ADC from post-RT mpMRI and PSA levels measured 6 months after RT offers a valuable prognostic tool.

These findings underscore the novelty of our study, not only demonstrating that both ADC and PSA can independently serve as prognostic factors following RT, but also suggesting that their combined use provides additive prognostic value. Specifically, the integration of a biochemical marker (PSA) and a radiological marker (ADC) enables the identification of a patient profile associated with poorer prognosis and an increased risk of progression and metastatic recurrence, compared to profiles characterized by an independently prognostic factor.

Although the individual predictive values of PSA and ADC have been previously investigated [3,5,6,8,9], to our knowledge, no study has assessed their combined prognostic power.

Regarding ADC as a prognostic factor, Liu et al. [8] reported that, in a retrospective study of 78 high-risk patients treated with RT and ADT, lower post-RT ADC values were significantly associated with local relapse (1.27 vs. 1.49 × 10^−3^ mm^2^/s, *p* = 0.001). Their ROC analysis showed strong discrimination ability (AUC = 0.88) with a proposed cutoff value of 1.34 × 10^−3^ mm^2^/s.

Similarly, Onal et al. [6] evaluated 229 patients with low- and intermediate-risk prostate cancer treated with RT alone and identified an ADC cutoff of 0.96 × 10^−3^ mm^2^/s, where patients below this threshold exhibited significantly worse 5-year biochemical relapse-free survival (85.5% vs. 100%; *p* < 0.001).

In our previously published study [9], we analyzed a cohort of 98 prostate cancer patients treated with RT and ADT, confirming that lower ADC values at 6 months post-RT were associated with higher rates of local relapse, biochemical failure, and disease progression. An ADC cutoff of 1.24 × 10^−3^ mm^2^/s effectively stratified patients for PFS over 10 years (85.6% vs. 58.6%; *p* = 0.004).

Regarding PSA, Kwak et al. [3] analyzed data from 7284 patients across 16 clinical trials and found that a post-RT PSA level ≥ 0.1 ng/mL was associated with poorer MFS, PCSS, and OS. Similarly, Naik et al. [11] demonstrated that 6-month post-RT PSA > 0.1 ng/mL was an independent predictor of worse outcomes across multiple survival endpoints.

Collectively, previous studies have shown that both ADC and PSA independently serve as predictors of treatment outcomes in prostate cancer. However, the integration of these two biomarkers had not been previously explored.

In our study, combining PSA and ADC values at 6 months post-RT enabled a more refined risk stratification, with patients with Profile C having lower PFS compared with patients with Profiles A and B, highlighting the additive prognostic value of combining both parameters. However, only nine patients in our cohort were classified as Profile C, underscoring the low frequency of this response pattern, characterized by both high PSA and low ADC values, following RT and ADT.

Metastasis-free survival (MFS) analysis further supported these findings, with Profile C patients exhibiting a 33.3% metastasis rate and significantly lower 8-year MFS compared to Profiles A and B, HR: 5.566 (95% CI: 1.119–27.692; = 0.047).

Identifying this subset of patients with a poorer prognosis could have significant implications for clinical management. These patients could benefit from closer follow-up strategies, including intensified PSA monitoring, mpMRI, or PSMA-PET imaging, as well as potential treatment intensification.

Therapeutic intensification for these patients could include the addition of androgen receptor pathway inhibitors to ADT, as in the STAMPEDE trial [12], or longer ADT treatment.

Our study has several limitations, including its retrospective nature and the limited sample size. Although the inclusion of only patients who underwent mpMRI at 6 months could introduce a potential selection bias, the institutional protocol at our center recommends performing post-radiotherapy mpMRI at the discretion of the treating physician. Nevertheless, findings from this study may support the routine use of mpMRI at 6 months after the completion of RT. Also, ADC measurements are operator-dependent, and although evaluated by experienced radiologists using a 3.0 T scanner (Signa HDxT-3T; GE Medical Systems, Milwaukee, WI, USA) mpMRI, some degree of variability cannot be excluded. This variability may influence the measurement of ADC values, potentially resulting in differing ADC readings and consequently affecting their prognostic significance. These considerations underscore the critical importance of standardized and carefully protocolized ADC value acquisition and timing across patients, emphasizing the need for consistent methodology and cautious interpretation. The small sample size of Profile C (nine patients) may limit statistical power, especially in multivariate analyses; therefore, these results should be interpreted with caution. Lastly, while our follow-up period is long, and this is the first study to assess the combined prognostic value of PSA and ADC, these findings should be confirmed in future studies with prospective designs and larger patient cohorts.

This study lays the groundwork for future research aimed at validating ADC in combination with PSA as a reliable prognostic biomarker and emphasizes the application of mpMRI following radiotherapy in prostate cancer patients.

## 5. Conclusions

Integrating ADC values and PSA levels at 6 months post-radiotherapy may be a valuable prognostic tool for treatment outcomes in prostate cancer patients undergoing RT and ADT. Patients with both high PSA and low ADC levels demonstrated significantly poorer progression-free and metastasis-free survival, identifying a high-risk subgroup for treatment failure. These findings underscore the clinical value of combining imaging and biochemical markers to enhance risk stratification and improve post-treatment management strategies.

## Figures and Tables

**Table 1:** Baseline characteristics of the patients included in the study.

	Profile A (n = 55)	Profile B (n = 34)	Profile C (n = 9)	*p*-Value
Age (mean ± SD)	71.7 ± 7.7	70.5 ± 5.9	70.0 ± 7.1	0.309
Initial PSA (median [Q1–Q3])	9.58 [6.90–18.00]	12.35 [6.67–25.97]	18.70 [9.50–75.00]	0.164
Initial ADC (median [Q1–Q3])	0.76 [0.66–0.90]	0.83 [0.69–0.99]	0.71 [0.69–0.79]	0.270
T Stage (n, %)				
T1–T2	26 (47.3)	17 (50.0)	8 (88.9)	0.065
T3–T4	29 (52.7)	17 (50.0)	1 (11.1)	
Risk group (n, %)				
Intermediate	10 (18.2)	11 (32.4)	4 (44.4)	0.125
High or very high	45 (81.8)	23 (67.6)	5 (55.6)	
Gleason score (n, %)				
Gleason ≤ 7	38 (69.1)	24 (70.6)	4 (44.4)	0.303
Gleason ≥ 8	17 (30.9)	10 (29.4)	5 (55.6)	

## Data Availability

All data generated or analyzed during this study are included in this published article.

## Abbreviations

ADT: Androgen deprivation therapy; AUC: Area under the curve; HR: Hazard ratio; LRFS: Local recurrence-free survival; mpMRI: Multiparametric magnetic resonance imaging; PCa: Prostate cancer; PFS: Progression-free survival; PPV: Positive predictive value; ROC: Receiver operating characteristic; RT: Radiotherapy; SD: Standard deviation; DRE: Digital rectal examination; ADC: Apparent diffusion coefficient; DW: Diffusion-weighted; NCCN: National Comprehensive Cancer Network; PSA: Prostate-specific antigen; CT-CAP: Computed tomography of the chest, abdomen, and pelvis; PET: Positron emission tomography; PSMA: Prostate-specific membrane antigen; Gy: Gray; EQD2: Equivalent dose in 2 Gy fractions; CTV: Clinical target volume; TR: Repetition time; TE: Echo time; FOV: Field of view; DW-MRI: Diffusion-weighted magnetic resonance imaging; NPV: negative predictive value.
